# Placental Peripartum Pathologies in Women with Preeclampsia and Eclampsia

**DOI:** 10.1155/2018/9462938

**Published:** 2018-09-20

**Authors:** Chijioke Ogomegbulam Ezeigwe, Charles Ikechukwu Okafor, George Uchenna Eleje, Gerald Okanandu Udigwe, Daniel Chukwuemeka Anyiam

**Affiliations:** ^1^Department of Obstetrics and Gynecology, Nnamdi Azikiwe University Teaching Hospital, PMB 5025 Nnewi, Nigeria; ^2^Fetomaternal Unit, Department of Obstetrics and Gynecology, Nnamdi Azikiwe University, Nnewi Campus, PMB 5001 Nnewi, Nigeria; ^3^Effective Care Research Unit, Department of Obstetrics and Gynecology, Nnamdi Azikiwe University, Nnewi Campus, PMB 5001 Nnewi, Nigeria; ^4^Department of Histopathology, Nnamdi Azikiwe University, Nnewi Campus, Nnewi, Nigeria

## Abstract

**Objective:**

To determine the pattern of pathological changes in placentas of preeclamptic/eclamptic parturients and its correlation with the clinical severity as well as the perinatal outcome.

**Methods:**

A cross-sectional analytical study of placental pathologies in preeclamptic/eclamptic patients was performed in a blinded pattern and compared with matched normal controls. Data were analyzed using Epi-Info 2008 version 3.5.1.

**Results:**

Placental pathologies were evaluated in 61 preeclamptic/eclamptic patients and in 122 controls. Of the 61 placentas, 53 (4.7%) were of preeclampsia while 8 (0.71%) were of eclampsia. Of the preeclamptic group, 14 (23%) had mild preeclampsia while 39 (63.9%) had severe preeclampsia. Infarction, haematoma, and some histological changes increased with the severity of preeclampsia (*p* < 0.001). When comparing placentas in eclampsia, severe preeclampsia, mild preeclampsia, and normal controls, there was respective increase in the presence of any infarction (75%, 66.7%, 35.7% vs. 12.3%) or any haematoma (100%, 100%, 71.4% vs. 35.2%), decidual arteriopathy (87.5%, 76.9%, 64.3% vs. 35.2%), cytotrophoblastic proliferation (75%, 71.8%, 42.9% vs. 25.4%), and accelerated villous maturation (75%, 69.2%, 57.1% vs. 31.1%). There was no statistically significant difference in placental calcifications, stromal oedema, stromal fibrosis, and syncytial knots. Degree of placental infarction was correlated with the fetal birth weight. The fetal birth weight with placental involvement of >10% was significant (*p*=0.01).

**Conclusion:**

In mild or severe preeclampsia/eclampsia, placentas had significant histological signs of ischaemia and degree of placental involvement by infarction is inversely proportional to fetal birth weight. While feto-placental ratio was higher with increased severity of the disease, the mean weight was less. This trial is registered with researchregistry3503.

## 1. Introduction

The triad of placenta, fetus, and mother continues to form a composite functional equilibrium during prenatal period, and dysfunction of any one of them can jeopardize the others. The dysfunction of the placenta often could lead to preeclampsia/eclampsia [1–5]. Preeclampsia and eclampsia remain the major causes of maternal and perinatal mortality and morbidity worldwide, causing 12–15% of direct maternal deaths [6, 7]. The incidence of preeclampsia is 2-0%, depending on the population studied; and definition of preeclampsia and age less than 20 years are significantly affected [8]. Although little is known about the pathogenesis of preeclampsia, recent study has revealed that deficiency of vitamin C, vitamin E, fat, zinc, and calcium, and excess of calories and carbohydrate were associated with increased risk of preeclampsia and eclampsia [9].

Thus, placenta has remained the most accurate record of the infants' prenatal experience. It is a unique and wonderful organ that arises de novo, and directly relates to the growth and development of the fetus in utero [10]. Being an organ of vital importance for the perpetuation of pregnancy and fetal nutrition, it has evoked great interest among obstetricians and pathologists as well, and there is a dearth of studies to understand the “unique biological status” of this complex organ in patients with preeclampsia/eclampsia [10].

The histological changes in preeclamptic/eclamptic placentas include infarcts, increased syncytial knots, hypovascularity of the villi, cytotrophoblastic proliferation, thickening of the trophoblastic membrane, obliterative enlarged endothelial cells in the fetal capillaries, and atherosis of the spiral arteries in the placental bed [11]. The root cause of preeclampsia is the placenta. Preeclampsia begins to abate with the delivery of the placenta and can occur in the absence of a fetus but with the presence of trophoblast tissue with hydatidiform moles. In view of this, the study of placenta should provide insight into the pathophysiology of preeclampsia/eclampsia [12].

Additionally, in recent years, it has been revealed that there is a clear relationship between placental pathology and intrauterine growth restriction/preeclampsia [13]. However, most of the studies so far on placental changes in preeclampsia and eclampsia were done on Caucasians and African blacks living in the western countries [11, 14–18]. Some of these studies have yielded contradictory findings [11, 13–18]. Apparently, the same pathological changes will be expected irrespective of the nationality. Therefore, there is need for an indigenous study to evaluate the placentas of these patients and develop indigenous database for patients' management. With increased litigation, it becomes imperative to reevaluate examination of placentas postpartum. This study will be a stepping stone for more studies on placenta changes in preeclamptics/eclamptics which will enable us build our data-based information for local and international references.

This study aims to determine the pathological changes of placentas from mothers with preeclampsia or eclampsia and correlate the findings with the clinical severity of the disease, placental weight, and birth weights of the newborn babies.

## 2. Methods

The study was a cross-sectional analytical study carried out from August 2011 to February 2012. It consisted of two groups of patients: the study group and the control group. Due approval was obtained from the Nnamdi Azikiwe University Teaching Hospital (NAUTH) ethics committee (Reference No. NAUTH/CS/66/vol.3/93; approval date, 27 August 2010). The study was registered at http://www.researchregistry.com with a clinical study registration number of researchregistry3503. The study groups were women who presented to the labour ward of NAUTH, Nnewi, with confirmed diagnosis of preeclampsia/eclampsia. The study group also included those patients who had preeclampsia/eclampsia but were booked for elective caesarean section for other obstetric indications. The diagnosis of preeclampsia/eclampsia was confirmed by sequential taking of relevant history, determination of blood pressure, and urinalysis.

The control group consisted of selected women presenting to the labour ward of NAUTH for their management of labour during the same period of recruitment of the study group. The control group were screened and found to be without hypertensive disorders of pregnancy but they were matched for age (±2 years), parity (±1), and gestational age (±2 weeks) with that of the study group. Placental specimens were taken from all cases and the histopathologist was blinded from the study.

The sample size of the study group was calculated based on a preeclampsia/eclampsia prevalence of 3.7% [19] reported recently from the study center. This was done using the statistical formula: *N *= *Z* ^2^ *PQ* **/** *D* ^2^, by Taylor [20], where *N* is the sample size and *Z* is the standard normal deviation for a normal distribution and was taken as 95% confidence interval which corresponds to 1.96. *P* is a maximum known prevalence of the disease, while *Q* is 1 − *p* (proportion of persons free from the disease) and *D* is the degree of accuracy or precision expected (*D *= 0.05). Substituting for the above formula and including 10% attrition gave a sample size of 61 women. However, in order to achieve increased precision of the study findings, (2 × 61) 122 cases of normotensive and nonproteinuric women were recruited as control.

Preeclampsia was defined as blood pressure (BP) equal to or greater than 140/90 mmHg with proteinuria on a catheterized urine specimen of at least 1+, or a clean catch midstream urine 1+ [19]. Mild preeclampsia was defined as BP of 140/90 mmHg and above, but less than 160/110 mmHg [21]. Severe preeclampsia was defined as BP of 160/110 mmHg and above with 2+ or more proteinuria, or presence of oligohydramnios, oliguria, intrauterine growth restriction, HEELP syndrome, pulmonary oedema, and epigastric pain. Eclampsia was defined as occurrence of convulsion in pregnant women after 20 weeks gestation in the absence of medical causes in a patient who was not a known epileptic [22].

We included pregnant women of gestational age ≥28 weeks, singleton pregnancies, and consenting pregnant women. Women of gestational age <28 weeks, multiple pregnancies, and parturient with diabetes mellitus were excluded from the study.

After consents were obtained, the patients in the study group were evaluated for preeclampsia and eclampsia. The patients were examined clinically for elevated blood pressure medical history, age, parity, last menstrual period, and calculating the gestational age using the first day of the last menstrual period. History of fit, headache, dizziness, blurring of vision, and upper abdominal pain and the bedside urinalysis for sugar and protein were also done. Subsequently, the patients were managed according to the labour ward and unit protocol. On delivery, the placenta was weighed after trimming the edges, and the APGAR score of the baby was also determined. Superficial fetal vessels were drained of all blood and adherent blood clots were removed from the maternal surface. The placenta was weighed on a calibrated digital device and examined for gross abnormalities. After weighing and examining the placentas, the whole placentas were placed into labeled plastic containers, containing 10% formaldehyde solution.

At the histopathology laboratory, a consultant histopathologist (blinded from the clinical outcome of each placenta) was concerned with the macroscopic and the microscopic examination of the placentas. In order to be able to reliably evaluate the villous maturation of the placenta, the pathologist was only informed about the gestational age.

The tissues were then stained using Haematoxylin and Eosin and viewed histologically. Histological variables evaluated were villous maturation, decidual arteriopathy, infarction, accelerated intervillous maturation, cytotrophoblastic proliferation, stromal oedema, stromal fibrosis, and syncytial knots. Other eventual findings were noted separately.

Descriptive analysis was done using Epi-Info 2008 version 3.5.1 (Epi-Info, Centers for Disease Control and Prevention, Atlanta, GA, USA). The results were analyzed further by using cross tabulation to identify the possible relationship between variables. Analyses were performed using trend statistics of Mantel–Haenszel test and Student's *t*-tests where appropriate to test for significant difference in observed proportion between the cases and controls. A probability value of <0.05 was considered significant.

## 3. Results

A total of 61 women out of 1120 labour ward admissions seen during the period of the study had preeclampsia/eclampsia. Fifty-three (4.7%) were preeclampsia while 8 (0.71%) were eclampsia. Of these women recruited in the study, 14 (23%) had mild preeclampsia and 39 (63.9%) had severe preeclampsia. The control group consisted of 122 women without preeclampsia/eclampsia.

Table 1 shows the age distribution of the patients in a relationship to the severity of the disease and the control group. There was no significant difference between the preeclampsia/eclampsia and non-preeclampsia groups in relation to age (*X* ^2^ = 9.038, *p*=0.70).

About half of the patients (43%) were primigravidae while only 3 (5%) of the patients were grandmultiparous. The mean parity of both the case and control groups was 1.3 ± 1.5 and 1.4 ± 1.5, respectively. There was also no significant statistical difference between the control and study groups in relation to parity (see Table 2 for trend statistics on Mantel–Haenszel test).

The mean gestational age (37 vs 38 weeks; *p*=0.51), mean birth weight (2.66 vs 2.79 kg; *p*=0.81), mean placental weight (0.41 vs 0.50 kg; *p*=0.69), and the mean feto-placental ratio (6.50 vs 5.58; *p*=0.21) between the cases and the controls, respectively, were as shown. The mean gestational ages in mild preeclampsia, severe preeclampsia, and eclampsia were 37, 36, and 34 weeks, respectively. The control groups also have similar mean gestational ages (38, 36, and 35 weeks, resp.).

The mean birth weight for patients with mild preeclampsia, severe preeclampsia, and eclampsia were 2.66 kg ± 1.63 kg, 2.58 kg ±1.61 kg, and 2.45 kg ± 1.57 kg, respectively, while those of the controls were 2.79 kg ± 1.67 kg, 2.75 kg ± 1.66 kg, and 2.75 kg ± 1.66 kg, respectively. There was no statistical difference in the mean birth weight between mild preeclampsia, severe preeclampsia, eclampsia, and the control groups (*t*-test = 0.262, *p*=0.81; *t*-test = 0.525, *p*=0.60; *t*-test = 0.414, *p*=0.68, resp.) (Tables 3 and 4, resp.).

The mean placental weights in the study group were 0.41 kg ± 0.64, 0.37 kg ± 0.61 kg, and 0.33 kg ± 0.57 kg for mild preeclampsia, severe preeclampsia, and eclampsia, respectively, while those of control were 0.50 kg ± 0.71 kg, 0.51 kg ± 0.71 kg, and 0.51 kg ± 0.71 kg, respectively. There was a marginal difference in the mean placental weight between the mild preeclamptic, severe preeclamptic, eclamptic, and the control groups, but not statistically different (*t*-test = 0.404, *p*=0.69; *t*-test = 1.046, *p*=0.30; *t*-test = 0.63 *p*=0.54) (Tables 3 and 4, resp.).

The fetal/placental weight ratio showed a marginal increase from mild preeclampsia to eclampsia but a similar ratio among the study groups. The mean ratio is 6.50 : 1, 6.97 : 1, and 7.42 : 1, respectively, for the study group and 5.58 : 1, 5.39 : 1, and 5.37 : 1, respectively, for controls.

The difference in the mean ratio between the severe preeclampsia, eclampsia, and the control groups was statistically significant (*t*-test = 2.636, *p*=0.001, and *t*-test = 2.170, *p*=0.04, resp.) but not between mild preeclampsia and control (*t*-test = 1.280, *p*=0.21).

The macroscopic (gross) features of the placentas were as shown in Table 3. Except for 6–10% infarction, there was a significant statistical relationship between the presence of infarcts in severe preeclampsia, eclampsia, and control groups (*p* < 0.001, for both), but the difference in mild preeclampsia and control groups was not significant, except for 6-10% infarction (*p*=0.009).

All the placentas in the severe preeclampsia and eclampsia groups had some degrees of placental haematoma. The difference in the presence (two pluses) or absence of haematoma between mild preeclampsia, severe preeclampsia, eclampsia, and control groups was significant (*p* < 0.001, for all).

The incidence of calcification in the normal placentas in the present study was 40.2% (*n*=49) while the overall incidence in the study group was 41% (*n*=25). Except for mild preeclampsia (*p* < 0.001), there was no significant statistical difference between the presence of calcification in the severe preeclampsia, eclampsia, and control groups (*p* > 0.05).

Table 4 shows the relationship between the degree of infarction and the birth weight. The degree of infarction is inversely proportional to the birth weight. There was a statistical difference between the placental infarction of >10% and the fetal birth weight (*t*-test = 2.17, *p*=0.03; *t*-test = 2.636, *p*=0.001).

The relationship between the clinical severity of the disease and the histological findings was as presented in Table 5 (Supplementary Material Plates 1 and 2). Only decidual arteriopathy, accelerated villous maturation, and cytotrophoblastic proliferation showed statistical significant on analysis between the severe preeclampsia, eclampsia, and the control groups (*p* < 0.05). None of the histological findings showed any statistical difference between the mild preeclampsia and control groups (*p* > 0.05).

## 4. Discussion

The placenta has been described as the mirror of the perinatal mortality. A glance at the literature reveals that the preeclampsia-eclampsia syndrome exerts its deleterious effects on the placenta. It was found in this study that low placental weight was related to the severity of the preeclampsia/eclampsia than in the control group. However, there was no statistical difference. The average mean placental weight in the preeclampsia/eclampsia group was 0.37 kg, while the average mean weight in the control group was 0.51 kg. This compares favourably with Pandit et al.'s [22] mean placental weight of 0.39 kg and 0.48 kg in the preeclamptic/eclamptic and control groups, respectively. The two Indian studies [23, 24] have come to similar conclusions.

There is a linear relationship between placental weight and birth weight of baby that can be expressed by feto-placental ratio. The fetal/placental weight ratio showed a marginal increase in the diseased group, which is statistically significant between severe preeclampsia, eclampsia, and control groups (*p*=0.01 and *p*=0.04). An overall mean ratio of 7.0 : 1 and 5.4 : 1 were obtained for diseased and control groups, respectively. This compares favourably with 6.04 : 1 and 5.87 : 1, respectively, in the study by Pandit et al. [22]. This has been observed when preeclampsia developed close to term. Due to placental insufficiency, fetal growth is affected.

About 25% of the normal-term placenta contained infarcts involving <5% of the placental parenchyma, but their frequency of occurrence is increased in preeclampsia and extensive infarcts being in 60-70% of patients with severe disease [25]. Placental infarct of >5% surface area is considered pathological and more frequently seen in preeclampsia due to thrombotic occlusion of maternal uteroplacental vessels [26].

In preeclampsia/eclampsia, 50% of placentas of patients have infarcts [25]; however, in this study, 36 (59%) of placentas of patients with preeclampsia/eclampsia had infarcts >5%. This is somewhat in comparison with 37% reported by Mirchandani et al. [27], 40.4% reported by Masodkar et al. [26], and 41% reported by Narasimha and Vasudeva [10] all in India. Only 7 (11.5%) of preeclamptic/eclamptic patients had infarcts covering more than 20% of the surface area of their placentas in this study. This is in distinct contradiction with the work of Vijay et al. [28] in New York, USA, where majority of patients had 50% of the surface area covered with infarcts. This may be due to the fact that the disease developed close to or at term. Early onset disease is associated with severe placental changes [26–28].

Minimal infarcts are not unusual findings in placentas delivered at term and are considered to be due to placental “aging.” In preeclampsia/eclampsia, however, there is accelerated “aging,” and widespread infarcts are common findings. There are, however, no histological or ultrastructural changes in the villi that can be considered as indicative of an aging process according to Fox [29].

Haematoma was a consistent finding in this study as all the placentas (100%) of severe preeclampsia and eclampsia had some degrees of haematoma. The difference in the presence of haematoma between the diseased and control groups was significant (*p* < 0.05). This is in contradiction with Moldenhauer's study in Ohio, USA, where he found haematoma to be the least common lesion in preeclampsia/eclampsia [30]. This disparity may be attributed to the preparation of the placentas after delivery. Moldenhauer washed the placentas before the pathological examination but was not so in this study.

Calcification is regarded as evidence of placental senescence or degeneration. The incidence of calcification in the normal placentas in the present study was 40.2% while the overall incidence in the diseased group was 41%. There was no significant statistical relationship between the presence of calcification in the diseased and control groups (*p*=0.380, *p*=0.830, and *p*=0.410, resp.). This is somewhat in agreement with the work of Narasimha and Vasudeva in Karnataka, India [10].

In the present study, the birth weight of baby was found to be decreased with increasing degree of placental infarction. Placental infarction >20% was associated with lower birth weight. There was a statistical difference between the involvement of >10% of placental surface and the birth weight (*p*=0.03 and *p*=0.01). This is similar to findings by Vinnars et al. [31] in Stockholm, Sweden.

Microscopic features observable under the light microscope include stromal oedema, stromal fibrosis, syncytial knots, cytotrophoblastic proliferation, accelerated villous maturation, and acute atherosis among others [5–8]. These changes were observed in more than 50% of the placentas that were examined.

This study demonstrated high frequencies of decidual arteriopathy, accelerated villous maturation, syncytial knots, cytotrophoblastic proliferation, stromal fibrosis, and stromal oedema in the preeclamptic/eclamptic group compared with the control group. Only decidual arteriopathy, accelerated villous maturation, and cytotrophoblastic proliferation showed statistical significant on analysis between the severe preeclampsia, eclampsia, and the control groups (*p* < 0.05). None of the histological findings showed any statistical difference between the mild preeclampsia and control groups (*p* > 0.05).

Decidual arteriopathy was the most consistent histological finding in this study; hence, Vijay et al. [28] considered it to be diagnostic of preeclampsia in New York. Even though decidual arteriopathy can be seen in parts of the placental tissue, which were studied, the best method of detecting it is by examination of placental bed biopsies, which was not accessed in this study. Therefore, the true frequency of decidual arteriopathy is probably considerably higher than was observed in this study.

Although hypertension is a requisite to diagnosing preeclampsia, absolute blood pressure alone is not always a dependable indicator of its severity. The histological findings in mild preeclampsia, severe preeclampsia, and eclampsia were similar; therefore, differentiation between mild and severe preeclampsia can be misleading because in clinical practice, a mild disease may progress rapidly to severe disease [4, 10]. Thus, some of the outcomes assessed were surrogate outcomes and could not be reflected in the sample size calculation used and we could not determine the body mass index. Since some of the comparators employed in this study were surrogate outcomes for comparison, we need to be critical while interpreting the findings of this study. Additionally, we could not assess any possible correlation between thrombosis of the fetal stem vessels and the prematurity of the baby or perinatal outcomes (whether preterm or postterm) and we could not provide a clearer pathological image due to authors' technical difficulties encountered. We could also not observe for basement membrane thickening, villous stromal fibrosis, perivillous and intervillous fibrin, fibrinoid necrosis and excessive syncytial knotting, extra villous cytotrophoblastic proliferation, and intervillous haemorrhage. These are subject to further study.

Fetal compromise is therefore generally the rule and perinatal mortality is high as reflected in a maternal mortality and a perinatal mortality recorded among eclamptics. If the mortality is expressed per number of eclamptic women, it will be one death for every eight eclamptics. It is obvious, therefore, that preeclampsia and eclampsia are major contributors to maternal and perinatal mortality in developed and developing countries [10, 18].

## 5. Conclusion

The placenta plays a central role in the origin and pathophysiology of preeclampsia. Here, it was demonstrated that a cardinal sign of placental hypoxia, that is, infarction, correlates to the clinical severity of the disease and fetal outcome, regardless of gestational age. The occurrence of infarction was more common and the amount of infarction was greater in severe preeclampsia and eclampsia in comparison to the control group. The correlation between the amount and extent of infarction and the clinical degree of symptoms indicates that mild preeclampsia, severe preeclampsia, and eclampsia are manifestations of different maternal response, rather than just preeclampsia forms depending on different gestational ages.

Preeclampsia/eclampsia adversely influences the weight of the placenta and fetal outcome. Thus, placenta acts as an effective index by examination of which we can predict the status of fetus in neonatal life as it can act as an indicator to the overall development of the fetus in preeclamptic/eclamptic cases.

## 6. Recommendation

Should all placentas be sent for detailed examination of placenta if there is adverse perinatal outcome; it is still not clear whether the pregnancy and neonatal outcomes are any different in abnormal placental histology group as compared with that of normal histology.

Guidelines suggest that all placentas should be examined by the trained perinatal pathologists. Placental pathology should be a routine component of obstetric-neonatal care, as it would provide detailed information, helpful in the postnatal management of adverse pregnancy outcome. However, the histological examination and perinatal outcome in most pregnancies is normal; for example, in the current study, the frequency of histologically normal examination was observed in over 50% of cases, even in the study group only few histological lesions were seen in the majority of the placentas. Therefore, the data from this study do not support the recommendations for the routine examination of placenta by the pathologist [32].

## Figures and Tables

**Table 1:** Distribution by age of patients for preeclampsia (PE)/eclampsia and control groups.

Age(YRS)	Mild PE, *n* (%)	Severe PE, *n* (%)	Eclampsia, *n* (%)	Entire study group, *n* (%)	Control, *n* (%)
<20	0 (0.0)	2 (50.0)	2 (25.0)	4 (6.60)	8 (6.60)
20–24	5 (35.7)	17 (43.6)	3 (37.7)	25 (41.0)	50 (41.0)
25–29	3 (21.4)	10 (25.6)	2 (25.0)	15 (24.6)	30 (24.6)
30–35	4 (28.6)	4 (10.3)	1 (12.5)	9 (14.8)	18 (14.8)
>35	2 (14.3)	6 (15.4)	0 (0.0)	8 (13.10)	16 (13.10)
Total	14	39	8	61 (100%)	122 (100%)
Mean age				25.5 ± 5.3	25.9 ± 5.7

*X* ^*2*^ = 9.038; *p*=0.6997.

**Table 2:** Distribution by parity of patients for preeclampsia (PE)/eclampsia and control groups.

Parity	Mild PE, *n* (%)	Severe PE, *n* (%)	Eclampsia, *n* (%)	Entire study group, *n* (%)	Control, *n* (%)	^*∗*^Trend statistics
0	5 (35.7)	17 (43.6)	4 (50.0)	26 (42.6)	53 (43.4)	0.916
1	3 (21.4)	7 (17.9)	2 (25.0)	12 (19.7)	23 (18.9)	0.895
2	3 (21.4)	4 (10.3)	1 (12.5)	8 (13.1)	16 (13.1)	1.000
3	1 (7.10)	5 (12.8)	1 (12.5)	7 (11.5)	15 (12.3)	0.873
4	1 (7.10)	4 (10.3)	0 (0.0)	5 (8.20)	9 (7.40)	0.845
>4	1 (7.10)	2 (5.10)	0 (0.0)	3 (4.90)	6 (4.90)	1.000
Total	14	39	8	61 (100.0%)	122 (100.0%)	

^*∗*^Mantel–Haenszel test.

**Table 3:** Distribution of gross placental changes for preeclampsia (PE)/eclampsia and non-preeclampsia groups.

Placental changes	Mild PE, *N*=14	Severe PE, *N*=39	Eclampsia, *N*=8	Study group, *N*=61	Control, *N*=122	Trend statistics of PE, SP, and eclampsia, resp.
	*n* (%)	*n* (%)	*n* (%)	*n* (%)	*n* (%)	
*Infarction (%)*						
0–5	9 (64.3)	13 (33.3)	2 (25.0)	24 (39.3)	107 (87.7)	0.020; <0.001; <0.001
6–10	5 (35.7)	6 (15.4)	1 (12.5)	12 (19.7)	13 (10.7)	0.009; 0.427; 0.871
11–20	0 (0.0)	15 (38.5)	2 (25.0)	17 (27.9)	1 (0.80)	0.735; <0.001; <0.001
>20	0 (0.0)	5 (12.8)	3 (37.5)	8 (13.1)	1 (0.80)	0.735; 0.001; <0.001

*Haematoma (%)*						
Absent	4 (28.6)	0 (0.0)	0 (0.0)	4 (6.60)	79 (64.8)	0.009; <0.001; <0.001
+	7 (50.0)	15 (38.5)	3 (37.5)	25 (41.0)	31 (25.4)	0.053; 0.117; 0.453
++	3 (21.4)	24 (61.5)	5 (62.5)	32 (52.5)	12 (9.80)	0.191; <0.001; <0.001

*Calcification(%)*						
Absent	8 (57.1)	24 (61.5)	4 (50.0)	36 (59)	73 (59.8)	0.846; 0.850; 0.585
+	2 (14.3)	13 (33.3)	3 (37.50)	18 (29.5)	45 (36.9)	0.093; 0.688; 0.972
++	4 (28.6)	2 (5.10)	1 (12.50)	7 (11.50)	4 (3.30)	<0.001; 0.597, 0.191

^*∗*^Mantel–Haenszel test.

**Table 4:** Relationship between degree of placental infarction and birth weight (BW).

Infarction (%)	Cases mean BW (kg)	Control mean BW (kg)	*p* value
0–5	2.85	3.01	0.820
6–10	2.61	2.72	0.120
11–20	2.53	2.65	0.030
>20	2.26	2.44	0.010

**Table 5:** Distribution of histological findings in relation to clinical severity of preeclampsia (PE) and non-preeclampsia groups.

Histological findings	Mild PE, *n* (%)	Severe PE, *n* (%)	Eclampsia, *n* (%)	Control, *n* (%)	*p* value of mild PE, severe PE, and eclampsia, resp.
Stromal oedema	5 (35.7)	24 (61.5)	2 (25.0)	55 (45.1)	0.500; 0.070; 0.460
Stromal fibrosis	7 (50.0)	19 (48.7)	3 (37.5)	38 (31.1)	0.260; 0.070; 0.990
Syncytial knots	7 (50.0)	17 (43.6)	5 (62.5)	35 (28.7)	0.120; 0.070; 0.070
Cytotrophoblastic proliferation	6 (42.9)	28 (71.8)	6 (75.0)	31 (25.4)	0.280; 0.001; 0.010
Accelerated villous maturation	8 (57.1)	27 (69.2)	6 (75.0)	38 (31.1)	0.100; 0.001; 0.001
Decidual arteriopathy	9 (64.3)	30 (76.9)	7 (87.5)	43 (35.2)	0.670; 0.001; 0.001

## Abbreviations

HEELP: Hemolysis, elevated liver enzymes, and low platelet count.

## Supplementary Materials

Supplementary Materials Plate 1: photomicrograph showing the placenta from the eclampsia group. Plate 2: photomicrograph showing the placenta from the severe preeclampsia group.
