# Supplementary material for: Placental Peripartum Pathologies in Women with Preeclampsia and Eclampsia
Source: Obstet Gynecol Int. 2018 Sep 20;2018:9462938. doi: 10.1155/2018/9462938 (PMC6171203; doi:10.1155/2018/9462938)
Supplement: Supplementary Materials — Plate 1: photomicrograph showing the placenta from the eclampsia group. Plate 2: photomicrograph showing the placenta from the severe preeclampsia group. [file 9462938.f1.zip › 9462938.f1/EZEIGWE-ELEJE-PLATE 2_OGI_2460664.docx]

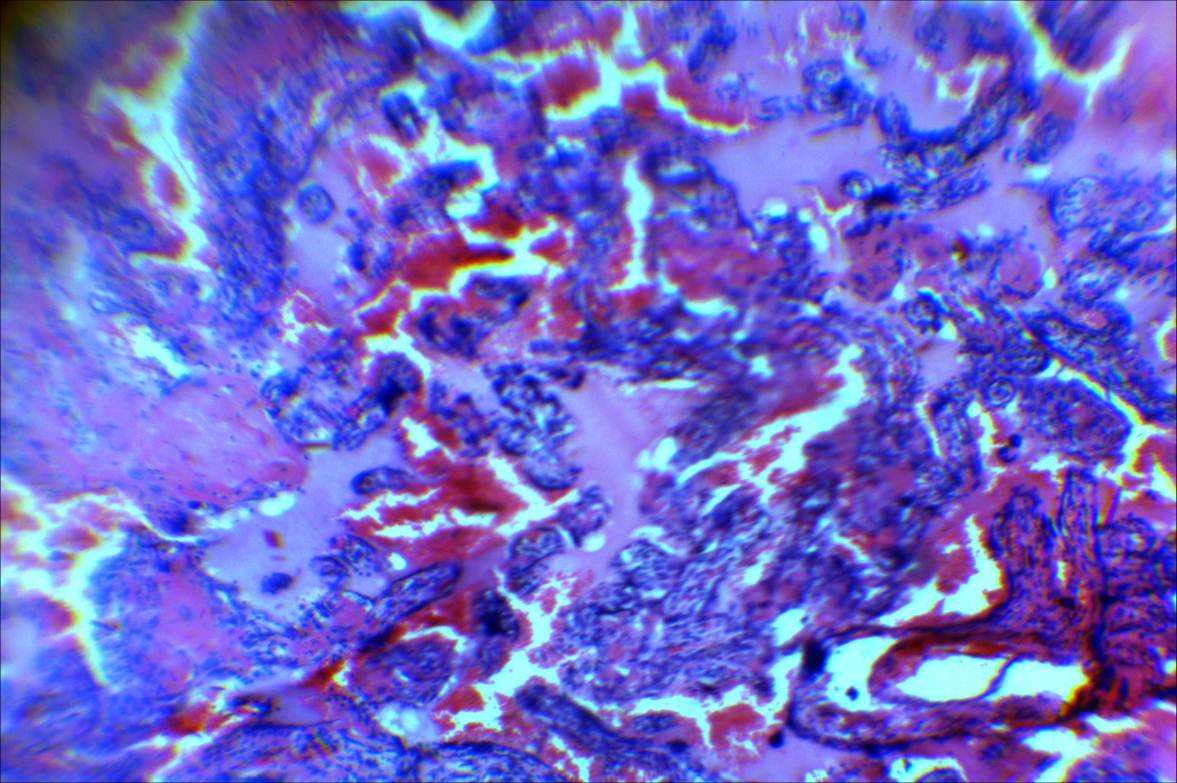


**Haemorrhagic necrosis**

**Infarctions**

**Stromal fibrosis**

**Decidual arteriopathy**

**Stromal oedema**

**Plate 2- Placenta from severe pre-eclampsia group**
